# Public authorities for transformative change: integration principle in public funding

**DOI:** 10.1007/s10531-023-02542-w

**Published:** 2023-03-10

**Authors:** Jerneja Penca

**Affiliations:** Science and Research Centre Koper, Mediterranean Institute for Environmental Studies, Garibaldijeva 1, Koper, Slovenia

**Keywords:** Sustainability transition, Integration principle, Biodiversity conservation, Do no harm, Integrated management, Transformative governance

## Abstract

Although science widely supports moving towards transformative change through integrating biodiversity into decision-making, and arguing for the essential role of public authorities, it falls short on suggesting specific means to that end. This article considers the EU’s approach to fostering the green transition as part of its post-pandemic recovery while exploring how the integration of biodiversity considerations could be integrated into decision-making. The rationale and implementation of the EU’s *do no harm* principle is examined, which functioned as a condition for public funds. The analysis shows the mentioned EU policy innovation has a very limited impact. The role of *do no harm* has been limited to validating, rather than initiating policy measures. It has failed to influence the design of measures such that they would benefit biodiversity and not encouraged synergies between the climate and biodiversity goals. Based on the experience with *do no harm* as well as the more focussed regulatory action directed at the goal of climate neutrality, the article lists key steps for fostering biodiversity integration in policy planning and policy implementation. These steps encompass substantive and procedural approaches and aim for deliberation, target-setting, tracking, verification and screening. There is considerable scope for robust regulation to play a role in support of the biodiversity goals alongside transformative bottom-up initiatives.

## Introduction

Scientific urge for a change in humanity’s development trajectories is starting to be translated into action. The nature’s unprecedented and dangerous decline (Rockström et al. 2009; Steffen et al. 2015; Bai et al. 2016) calls for ‘a fundamental, system-wide reorganization’ (IPBES 2019) across psychological, behavioural, social, cultural, economic, political, governance, institutional, demographic, technical and technological factors and changes (IPBES 2021). In the past years, the scientific appeals appear to start being picked up in political commitments to action. Governance frameworks at various levels have made a transformative change a signpost for their action (UN GA 2015; European Commission 2019; CBD 2021). Policy attention to transformative change was particularly strengthened by the Covid-19 pandemic, as this revealed societal vulnerabilities in face of globalization, social inequities, unsustainable production and consumption patterns, and ill-prepared governance (United Nations 2021), and triggered an unprecedented social energy for redesigning sustainable relationship between people and nature (McNeely 2021).

However, the operationalization of transformative change remains challenging. The literature on transformative change is growing, but it is heavily dominated by talking about transformation (“the blah blah of transformation”), rather than outlining how we can move toward action for results (Bentz et al. 2022). In part, this is due to the incredible complexity of manging a societal transition among multiple and linked biophysical and social interactions and constraints. Contemporary socio-ecological systems are subject to rapid and unprecedented change, multiple drivers, dynamic and unpredictable structures, unknown phenomena and unintended consequences (Bai et al. 2016; Reyers et al. 2018). Add to that the density of today’s governance landscape, which involves a myriad of actors, from state to non-state, acting at multiple levels and with a heavy interdependence (e.g. Manning and Reinecke 2016). But in part, the lack of details on the potential pathways towards the realisation of transformative change is also because there has been little assessment of the concrete governance efforts that tried to introduce such a change.

The question of the type of governance in support of transformative change at the intersection of societal and environmental challenges has been discussed since early considerations on transitions to sustainable development (Grin, Rotmans, and Schot 2010; Frantzeskaki et al. 2012). It was accepted that transitions consisted of strategic planning, operational work and reflective learning spanning over a long term (Loorbach 2010). Gradually, a consensus emerged around contemporary socio-ecological challenges requiring a fundamental reorganisation of institutions and governance approaches (Berkes 2017; Stout and Love M. 2020; Turnheim et al. 2020). While discussing governance *for* and *of* transformations, it became clear that also transformation *of* governance is at stake (Chaffin et al. 2016; Burch et al. 2019).

Governance of transformative change is now recognised as a systems-effort, encompassing a variety of bottom-up actions from communities and top-down steering from public authorities. Both are needed—tangible differences at the level of individuals and local communities, but also systemic transformations in how humans as societies relate to the planet and to each other at the organisational level. Indeed, finding a balance and coordinating between these two directions and levels remains a key dilemma in effecting transformation (Frantzeskaki et al. 2012; Pereira et al. 2020). However, the literature has not divided the attention equally among the two levels or sources of change. While empowerment actions initiated by local communities and civil society towards transformations are examined rather widely (Trutnevyte et al. 2011; Fröcklin, Jiddawi, and de la Torre-Castro 2018; Andrachuk et al. 2018; Penca 2019; Colloff et al. 2020; Zwarteveen et al. 2021; Shi and Moser 2021), explorations of governments as actual frontrunners of sustainability actions has been considerably more scarce (Willi et al. 2020). The discussion about the role of public authorities (states, governments) in the context of transitions towards sustainability has remained at the theoretical level (Johnstone and Newell 2018; Eckersley 2021; Bolton 2022) and in form of appeals that they become stronger actors in transformations (Mazzucato 2015; Deutz and et al. 2020). Portrayals of them actually being in the driver’s seat of transformations have been rare.

If governments are to administer transformations of social and economic components away from destructive pathways, knowledge on what does or does not work is needed. Indeed, such instructions are sought by the policy-makers (Turnheim et al. 2020). The kind of knowledge needed goes beyond describing broad types of governance approaches, and has to include links to specific policy tools. ‘Transformative governance’ has been widely advocated for (Chaffin et al. 2016; Patterson et al. 2017; Burch et al. 2019; Linnér and Wibeck 2021; Visseren-Hamakers et al. 2021). However, these ideas remain short of suggesting concrete policy tools and actions for triggering, steering, accelerating and maintaining the type of shifts.

One important policy approach is the recommendation of integration of policies (Visseren-Hamakers 2015; Stafford-Smith et al. 2017; Turney et al. 2020; Pettorelli et al. 2021; Pörtner et al. 2021; Plank et al. 2021), dubbed also as policy coherence (Nilsson et al. 2012; European Commission, Joint Research Centre 2019; Pettorelli et al. 2021), nexus approach (Boas et al. 2016; Gupta et al. 2017; Pascual et al. 2022), or the idea of synergistic measures tackling simultaneously climate change and biodiversity (Hu, Chen, and Wu 2021). While widely commended as a cost-efficient option to jointly address the biodiversity and climate change crises, little is known about ways of improving it and scaling it up beyond what has evidently not worked so far.

The aim of this article is to strengthen the knowledge about policy approaches for transformative change and particularly the integration efforts, through the analysis of a concrete experience involving strategy-setting and regulation. The article looks at the approach adopted by the European Union (EU) in enacting ‘green transition’, declared as an ambitious political agenda. The EU is studied as a case of a governance framework with a high level of willingness to introduce systemic changes by public authorities. The EU has continually sought to assume leadership in environmental domain (Grubb and Gupta 2000) and aimed high in this ambit. The EU has strong constitutional ambitions (TEU Art 3 commits the EU to work for the well-being of its people as well as for sustainable development of Europe and in the world, a high level of protection and improvement of the quality of the environment). Moreover, its latest growth and development agenda (European Green Deal, EGD) identifies climate and environmental-related challenges to be of highest strategic importance and ‘this generation’s defining task’, justifying a set of ‘deeply transformative policies’ (European Commission 2019). The EU’s willpower for a systems’ overhaul, espoused by the EGD, and the rhetoric of tackling various environmental challenges in an interrelated manner, present a good starting point for studying policy options for transitions and reflecting on their success or failure. Although the EU is a political subject *sui generis*, its role is associated with that of a public authority and likened to that of the government or state for the purpose of this analysis, due to the regulatory and financial competences in this specific case.

The object of analysis is the EU’s experience with designing and implementing the post-Covid-19 recovery plan Next Generation EU (NGEU), a large stimulus package, aimed at repairing the economic and social damage inflicted by the pandemic in a way that builds a green and digital future (European Commission 2020c). We review the setup of the plan from the point of view of the implementation tools for transformative change. One of the central tools for implementation of the EU’s green transformation was the introduction of a new policy tool, a so called *do no harm principle*. Its rationale (guided by the objectives of life-cycle assessment and policy coherence) and its implementational prospect (its full respect being a condition for allocation of funds) renders the principle an important policy innovation for the implementation of the acclaimed integration principle. The analysis evaluates the potential and practice of this policy instrument through the review of knowledge about regulation of complex societal challenges and literature on transformations as well as an original empirical study. The article asks, whether *do no harm*, as propounded and implemented at the EU level, is conducive to a systemic reorganisation.

Contrary to the literature on transformative governance, where the analysis often remains on a fairly abstract level, this real-life example of a specific policy approach or management action, helps us understand the challenges of translating the guiding principle of ‘integration’ into action. But it also helps us see opportunities in using regulation for inducing sustainability transformations at the public policy level. The study foregrounds regulation as a tool for transformative governance and offers a better understanding of potential intervention points and methods.

The article proceeds as follows. In Section “Transformative change through public policy-making", the background to the case study is provided by revising knowledge regarding the role of public authorities in governing for sustainability (2.1), regulatory instruments available to governments (2.2.), and the meaning of integrative governance with regards to biodiversity benefits (2.3). In Section “Approach and methods", the approach and method to analysing the EU’s experience as an instance of an attempted transition are outlined. This is followed in Section “Results" by the presentation of results of the *do no harm* procedure, and in Section “Discussion: do no harm as a viable policy innovation?" by the evaluation of results in the context of knowledge from regulation, environmental policy and sustainability literature. Finally, the conclusion highlights public authorities and their regulatory task as holding a considerable potential for accelerating and shaping transformative change through shaping procedures and mandating targets in support of scientifically supported goals.

## Transformative change through public policy-making

What do we know about the ability and role of public authorities in introducing transformative change? What instruments can they use? Why is biodiversity mainstreaming key for sustainability transitions and how can it be achieved? This section combines key lessons from the existing literature on public policy, regulation, sustainability governance and transformations.

### Role of governments in complex times

Although rarely brought into a direct dialogue, both literature on regulation and transformative change relate to the question of governance in complex times, composed of multiple interactions and typically transcending sectors and jurisdictions. It has been suggested that transition literature conceptualises government in a way that is at odds with the traditional tasks of the government and public administration (Braams et al. 2021). But in fact, the expectations of the governments’ tasks in the context of sustainability challenges fit well with the account of the government’s role in a post-modern, polycentric, dynamic, complex environment—the context that has been at the focus of the regulation literature since 1990s. Here, the governments’ competences and assignments are understood as an ongoing, adaptive process, constantly changing and expanding for new tasks, designing moves and counter-moves through regulation (Ayres and Braithwaite 1992; Gunningham and Grabosky 1998). Regulation is understood in a great variety of ways (Baldwin, Scott, and Hood 1998; Morgan and Yeung 2007; Koop and Lodge 2015), broadly relating to a set of direct and intentional interventions, encompassing binding standard-setting, monitoring, and sanctioning, and exercised by public-sector actors on the economic activities of private-sector actors (Koop and Lodge 2015). We extend it to spanning over not only the level of legislators but also public administration (Croley 1998; Kingsbury et al. 2004). In the transnational landscape, regulation by states (but also by international organizations) is likened to orchestration or steering actors in desirable directions, both through binding rules and facilitative actions that encourage rather than mandate (Abbott and Snidal 2009).

According to the literature on transformations, the government’s role in transformation ranges from softer to harder methods. The state provides a platform for constituting, defining and redefining democracy, power and culture, from which norms, expectations, and beliefs emerge (Hausknost and Hammond 2020). It is capable of proposing and deliberating transformation (O’Brien 2012), through introducing, monitoring and adjusting policies and laws related to the management of the environment and societal–environmental interactions (Duit et al. 2016; Eckersley 2021). The government is also able to scale up and accelerate the changes introduced by individuals and communities (van Bruggen et al. 2019), and set up collaboration and co-creation arenas for various stakeholders and steer them in a broadly similar direction (Johansson 2018). The government’s role in introducing change is particularly strong through channelling public spending and conditioning it, especially in a post-crisis period (Mazzucato 2015; Spratt 2015).

The regulation and sustainability literature both agree that contemporary regulation requires action not only in response to challenges, but also in their anticipation. Regulation should not just be reactive, including deploying reflexivity and adaptive learning processes (Dryzek 2014; Galaz et al. 2012; Driessen et al. 2012; Berkes 2017), but increasingly also anticipatory, pre-emptive, forward-looking and acting in advance of risks (Chapin et al. 2010; Park et al. 2012; Boyd et al. 2015; Vervoort and Gupta 2018). Decision-making is required to be based on scenarios or models, and ideally occurring at the intersection of challenges, requiring a cohesive or ‘nexus’ approach (J Gupta et al. 2013).

### Regulatory instruments for transformative change

While the government’s roles and their broad approaches to introducing transformative change are justified, it is more difficult to determine the more specific methods or regulatory instruments to that end. Traditionally, in dealing with the socio-environmental domain, governments’ options range across direct regulation (so-called command-and-control), market-based incentives and information-based tools (incl. awareness-raising and behaviour nudges) (Jordan et al. 2011; Penca 2020). In the context of transformations, there is a prior and broader question to just picking the right tool in the regulatory toolbox. The regulatory goal here is new, wide-ranging, profound and only partially (pre)defined, in contrast with more narrowly defined goals, such as waste reduction, minimizing pollution, or habitat protection.

Rather than in terms of regulatory instruments, transformative change has thus been discussed foremost in the context of systemic socio-political changes. At the heart of these is the question of addressing power asymmetries and inequalities (Avelino 2017; IPCC 2022). This spans over the need to re-establish normative hierarchies in such a way as to re-embed economic policies and activities into social and environmental norms (Hujo, Braumann, and UNRISD 2016, 226) as well as over the need for more participatory decision-making approaches (Glass and Newig 2019; Cattino and Reckien 2021). Collective action among all actors is needed to foster fundamental change, both in making decisions and creating opportunities (Monkelbaan 2019). Transformative governance needs to be integrative, inclusive, informed and adaptive at the same time (Razzaque et al. 2019), while highly sensitive to the local context (Bennett et al. 2016; Andrachuk et al. 2018).

Due to uncertainty, governance for transformative change has been strongly linked to experimentalism and innovation (Moberg et al. 2021). Innovation in policy can be conceived in terms of introducing new regulatory elements, expanding their use into new areas or experiencing new effects (Jordan and Huitema 2014). Using a variety of approaches or policy mixes has been promoted (Rogge and Reichardt 2016; Edmondson, Kern, and Rogge 2019; Kern, Rogge, and Howlett 2019). But innovation in policy-making can also be conceived in terms of selecting different and better leverage points for overcoming the lock-ins that contribute to existing inertias (Abson et al. 2017; Kanger, Sovacool, and Noorkõiv 2020). Improvements can thus be of infrastructural and technological, institutional or socio-economic, behavioural and relational types, and all tightly intertwined among themselves (Seto et al. 2016). They can also be thought of in terms of restructuring institutions, reconnecting people to nature and Earth, and rethinking how knowledge in pursuit of sustainability is produced and used (Abson et al. 2017; Delanty and Mota 2017). Concepts of growth, efficiency, state, commons and justice were demonstrated the key to the transformation (McPhearson et al. 2021). Thus, transformative policy tools seem to start with the task of reframing policy goals.

Empirical evidence confirms that governments have a powerful role in transformations by devising the right public policies and in channelling public financing, but have to work in close collaboration with the private sector (Moberg et al. 2021). They have the ability to act quickly and decisively if they decide so (Willi et al. 2020). However, it is suggested that the governments should not approach transformative change as a managerial issue and in a problem-solving manner, but rather maintain a strategic and responsive focus on it (Bornemann and Christen 2020).

### Integration of biodiversity in support of transformation

A key concept with regards to operationalisation of transformation towards sustainability, is integration. Integration or the idea of linking various goals and pursuing multiple targets concurrently and not prioritising one over another, is central to Sustainable Development Goals (UN GA 2015), where the Preamble of the 2030 Agenda speaks of the 17 SDGs and 169 targets being ‘integrated and indivisible’ and where a separate target (17.14) aims to ‘enhance policy coherence for sustainable development’. The purpose of integration or policy coherence efforts is that rather than policies resulting in tensions and trade-offs among economic, social, environmental and governance dimensions, policies should result in coherence and multiple benefits at all stages of domestic and international policy making (OECD 2015; Kanie and Biermann 2017; Stafford-Smith et al. 2017). Indeed, the SDGs are designed in this interrelated way as numerous targets can contribute to several goals (ICSU and ISSC 2015). A close integration between social and environmental goals has indeed become well established in the holistic concept of socio-ecological systems (Díaz et al. 2015; Biggs, Schlüter, and Schoon 2015).

Recently, science has been drawing attention to the utmost need for synergies among different environmental goals and specifically to the paramount role of the biodiversity. Biodiversity loss, reflecting complex interactions between ecosystems and societal vulnerabilities, has been recognised as typifying the existence of a gap between existing and sustainable pathways (Palomo et al. 2021; Pörtner et al. 2021; Turnhout et al. 2021; Rusch et al. 2022). The efforts in biodiversity conservation are seen as the driver of profound shifts in society and sustainability transformations (Pascual et al. 2022), particularly when they are related to embracing other global challenges and increasing conservation and restoration ambitions (Leclère et al. 2020; Fastré et al. 2021; Jung et al. 2021; Leadley et al. 2022). The interconnected nature among biodiversity, climate and people has been acknowledged in the recent international policy processes and agreements, such as the 2015 Paris Agreement, the 2015 Sendai Framework for Disaster Risk Reduction; the post-2020 Global Biodiversity Framework and 2021 Glasgow Climate Pact.

Enacting transformative governance is thus closely related to the challenge of integration of biodiversity considerations into policy decisions at all scales and sectors. The requirement of environmental policies being an integral part of development is long established (Principle 4 of the Rio Declaration 1992), but also a concept capable of being implemented in various ways and to various degrees (Persson 2004). At the basic level, the principle of integration can act as a requirement at the procedural or outcome level (Barral and Dupuy 2015). This would suggest a difference between biodiversity considerations being simply taken into account in the process of decision-making, or representing a certain expectation of result. In practice however, successful cases of environmental integration are about both these dimensions (Lenschow 2002). Any discussion of integration principle in public policy is far from a discussion between prescription vs. inclusiveness. Public participation and engagement is an inherent element of both the integration principle (Lenschow 2002) and successful transformative governance led by the public authorities (Cattino and Reckien 2021).

Policy options have been discussed in the context of decision-making for the benefit of biodiversity conservation and sustainable use. Biodiversity can be addressed through a number of measures and as part of policy mixes in sector-specific ways, relating to food, energy and urban planning, to name just a few sectors (Razzaque et al. 2019). Literature has recognized the value of biodiversity being integrated into other policy actions and embraced by other global challenges, particularly climate change adaptation and mitigation (Morita and Matsumoto 2018; Leclère et al. 2020; Jung et al. 2021; Hu, Chen, and Wu 2021; Fastré et al. 2021; Leadley and et al. 2022). Independently of the need for creating synergies between policies for the benefit of biodiversity, convincing arguments have been made for increasing conservation and restoration ambitions (Díaz et al. 2020; Leclère et al. 2020; Leadley and et al. 2022) and requiring increasing resources dedicated to conservation (Deutz and et al. 2020; Grumbine and Xu 2021). The idea of increased investments into biodiversity can translate into either direct funding for biodiversity, elimination of subsidies with adverse impacts on biodiversity and into setting-up public works programmes with socio-environmental objectives (Hujo, Braumann, and UNRISD 2016). A fundamental reorientation of economies away from continuous growth and a reduction of overall resource consumption has been identified as a necessary means to reducing the overall pressure on biodiversity (Otero et al. 2020).

**Fig. 1:**
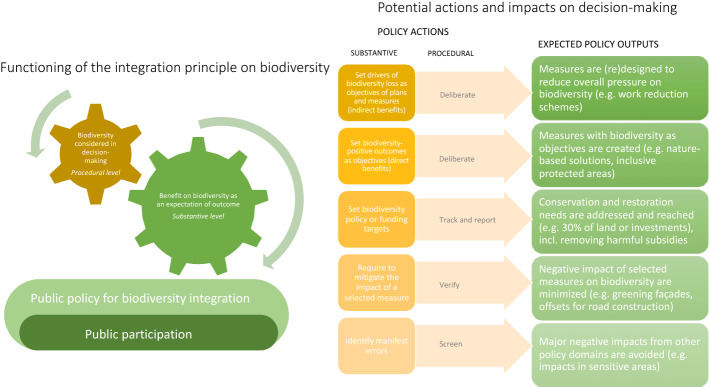
Functioning of the integration principle on biodiversity with possible policy actions and outcomes for biodiversity

Various policy options emerge from considering the integration principle at the intersection of regulatory theory and sustainability literature. The knowledge can be structured into a set of possible regulatory interventions for public authorities. The starting point for the list is a recognition that the principle of integration can be applied in various degrees, rather than in a yes/no manner. It allows and requires not only avoiding conflicts and trade-offs that originate from other policy domains or sectors, but also proactive planning and implementation of policies. Procedural and substantive approaches play a complementary role as they can influence policy-making through different methods (actions) and in different ways (outputs). Different substantive obligations are matched by different procedural approaches, and vice versa. Figure 1 summarises the model. The purpose of the figure is not to detail policy actions, but to present the key interventions at disposal of the public authorities in integrating or mainstreaming biodiversity policies.

## Approach and methods

To explore how integration of biodiversity as a means of transformative change is taking place, we analyse the EU’s approach to designing and implementing the transformation of its economy. We focus on the post-Covid-19 recovery plan, which brought about significant amounts of money to implement the EU’s internal strategy European Green Deal, adopted in 2019 (European Commission 2019). The regulation process is examined and evaluated from the point of view of promoting biodiversity-positive outcomes.

### The context to the EU’s sustainability transformation

In the EU the principle of integration, as a means to sustainable development, is enshrined in its constitutional treaty (Treaty on the European Union, Art 11): “Environmental protection requirements must be integrated into the definition and implementation of the Union’s policies and activities, in particular with a view to promoting sustainable development.” The principle has guided the design of specific policy instruments, such as Strategic Environmental Assessment to guide the plans and programmes that are likely to have significant effects on the environment (Directive 2001/42/EC), as well as the design of policies, for example an integrated maritime policy (e.g. Commission of the European Communities 2007). However, the operationalization of integration is not without barriers. The integration of environmental considerations into all sectors is hampered by the fragmented institutional design at various governance levels, from global to the EU and national (Doelle 2009; Russel et al. 2018). The EU is struggling particularly with renegotiating economic interests and beliefs, and overcoming institutional competition and sectoral policy boundaries (Winkel and Sotirov 2016).

In 2019, following the formation of a new Commission (proponent of legislation and the guardian of the EU’s legal order), a new European strategy was adopted, European Green Deal or EGD (European Commission 2019). In comparison to the previous Commission and its agenda, this one was considerably more environment-oriented (Čavoški 2020). This has laid promising ground for reforms in the direction of sustainability pathways, such that these would correspond to the expectations by the public across the EU, as expressed in the polls (European Commission 2020a). The EGD promised to design “a set of deeply transformative policies” across all the sectors, listing the economy, industry, production and consumption, large-scale infrastructure, transport, food and agriculture, construction, taxation and social benefits. Apart from planning a carbon–neutral economy by 2050, it represented a strong commitment to creating a non-polluting and non-toxic circular economy and to biodiversity goals and targets. The latter included recognizing the value of protecting and restoring natural ecosystems, and sustainable use of resources as well as their strong link to improving human health. It acknowledged issues as closely interlinked and mutually reinforcing, thus having a strong case for integration.

To consolidate the commitments from the strategic document of the EGD, the Commission embarked on a process of adopting action plans, laws and policy guidance. While slowed due to the Covid-19 pandemic, this resulted in a variety of policy measures, including an EU-wide climate law, making the EU’s climate neutrality by 2050 legally binding, an action plan for circular economy, a strategy for the transformation of the food system, development of a sustainable blue economy and a biodiversity strategy.

The EU Biodiversity Strategy for 2030 (adopted in May 2020) committed to legally binding restoration of degraded habitats, ecosystem services and the biodiversity, and to providing financial support for restoring ecosystems (European Commission 2020d). Considering that legal targets on biodiversity have long gone unnoticed, the early signs of a legislative action in this domain were applauded, particularly from the point of view of integrating biodiversity conservation effectively into other sectoral policies and scaling up the funding (Hermoso et al. 2022).

With the outbreak of the Covid-19 pandemic, and the decision that the EU will help tackle the economic consequences by large injections of money, the significance of the EGD was elevated. From a strategic document outlining legislation, EGD became the signpost for a post-pandemic transition, centred around the priorities of a green and digital Europe (European Commission 2020e). The EU availed unprecedented 672,5 billion Eur (312,5 billion Eur in non-repayable funds and 360 billion Eur in loans until 2027) to member states (Regulation 2021/241). Most of this funding was to be channelled to member states as either grants or loans. The payment of the funds to member states were conditioned on a plan (National Recovery and Resilience Plan, NRRP) outlining the public investments, and their compliance with the agreed rules. One of the agreed rules was compliance, in the adopted NRRPs, with the *do no harm* principle, which aimed at integration of environmental policies across the investments from this fund.

### Method of analysis

The role of the integration principle in the transformation was explored through the operation and impact of the *do no harm* principle. This principle was introduced in the EU as a means for verifying environmentally sustainable economic activities, including the spending of public money. In the present study, we first explore the setup of the principle, its intervention logics or theory of change, as explained in the publicly available working documents by the European Commission (the Commission). Second, we analyse the operation of the principle through a revision of Commission’s assessments of 24 NRRPs (out of 27 EU member states, 2 NRRPs were not approved by the time of writing and 1 had not been submitted). The application of the principle is observed through a combination of a qualitative analysis of documents regarding how biodiversity is mainstreamed into the NRRPs and quantitative analysis of the share of resources dedicated to biodiversity as a share of total resources, including conservation and restoration ambitions (at land or sea). The NRRPs did not specifically classify values for investments into biodiversity, neither was this done by two large-scale external reviews of the NRRPs.^1^ Information was extracted through the present original empirical analysis.

All reviewed documents (regulations, technical documents, Commission’s assessments of NRRPs) are publicly accessible. The Commission’s assessments of NRRPs were reviewed rather than NRRPs so as to mainstream the object of study from the linguistic and statistical point of view (not all NRRPs are available in English; they do not use a common approach to tagging and tracking investments).

## Results

### Rationale for do no harm principle

The *do no harm* principle builds on an existing terminology but represents an innovation from the point of view of the context and way in which it was used. A *do no harm* principle (or rule) is a widely recognized principle of customary international law, which bestows a responsibility on every State to prevent, reduce and control the risk of environmental harm to other states (Crawford 2019). The principle has been incorporated in international treaties, such as UN Convention on Law of the Sea, Convention on Biological Diversity and UN Framework Convention on Climate Change.^2^ However, from the obligation to not cause transboundary harm, the principle gained a different meaning in the context of the EU, where it is mandated with not causing harm to other policy areas, and thus aligned more closely with the principle of integration and coherency.

*Do no harm* also builds on a previous policy experience in the EU with insufficient integration of biodiversity and sustainability, particularly in the planning of energy policy. Concerns have been raised over the implications of the large-scale diffusion of renewable energy on ecosystems and biodiversity, as this becomes the backbone of transitioning to green economy (Gasparatos et al. 2017). Additionally, there is a history of the EU efforts having led to highly controversial measures, such as curbing greenhouse gas emissions in the transport sector by promoting crop-based biofuels with severe negative impacts on ecosystems and food production (Directive 2003/30/EC—Biofuels directive) or including forest biomass in the EU’s renewable energy directive (Directive 2018/2001; see Catanoso 2021). Member states indeed had a “strong sense of ownership of the energy and climate transition objectives”, but less for their interaction with other environmental goals (European Commission 2020f).

The EC first introduced the *do no harm* principle, in an undetailed way, in 2020 as part of the plan setting out an increased climate ambition of the EU, advocating that *do no harm* avoids “wasted money and stranded assets” (European Commission 2020b). The principle was further elaborated in documents relating to the post-Covid-19 Recovery and Resilience Facility Regulation. It is referred to in two versions—as *do no harm* (European Commission 2020b) and *do no significant harm* (Regulation (EU) No 2020/852, European Commission 2021a), without implying a difference in that distinction. The principle was presented as a new rule, based on the logics of life-cycle assessment. It applied to any economic activity that is to qualify as environmentally sustainable, including those that wished to benefit from the funds from the Recovery and Resilience Facility. Any economic activity (reform or investment) must “do no harm” to any of the six environmental objectives: climate change mitigation, climate change adaptation, sustainable use and protection of water and marine resources, circular economy, pollution prevention and control, and protection and restoration of biodiversity and ecosystems. Although the details on the implementation of the principle came only two months before the deadline for the submission of NRRPs (European Commission 2021a), the *do no harm* became a mandatory part of every NRRP. For every investment or reform, proposed to receive funds from the EU recovery fund, the national authorities had to evaluate the impact of that measure on the six indicated areas by writing, on the provided template, a yes/no indication of the impact and a brief justification. The national proposal could only be approved if no measure within the entire document was found to lead to significant harm to any of the six environmental objectives.

The *do no harm* principle was designed as the sole and crucial measure of the recovery process to support reforms and investments in “green transition, including biodiversity” and for mainstreaming biodiversity action in EU policies (Regulation (EU) 2021/241). In design, it was limited to being a plain procedural requirement with no participatory measures and no obligation of outcome. This occurred through a mal-devised legislative manoeuvre. While all investments had to contribute to one of the six pillars, among which was also ‘green transition’, which included biodiversity, biodiversity was not supported by a numerical target, unlike climate action (Regulation 2021/241). Contrary to the objective of climate neutrality and digital transition, which were supported by the numerical targets (of having to benefit from at least 37% and 20% respectively of the all the financial allocation, in each national plan), biodiversity had no comparable obligation of outcome. Furthermore, biodiversity-positive measures were not specifically encouraged. Biodiversity was not framed as one of seven ‘flagship areas’, which were strongly encouraged by the Commission, based on the fact that they are common to all member states, need significant investments, create jobs and growth and are needed for the transition (European Commission 2021b). The underlying logics of the *do no harm* principle was purely that a requirement for a self-revision of decisions would improve compatibility between the proposed investments and their impact across environmental areas.

### Application of the principle

The *do no harm* principle has been reported to have been consistently observed. All the NRRPs stated compliance with the principle for all measures, and in the Commissions’ assessments this was confirmed without exception. The justification for compatibility with the principle varied to some extent both across countries and across measures within the same country, ranging from brisk assurance of compatibility to somewhat longer simple justification. The varying answers reflect also a varying maturity of the NRRPs and individual measures in terms of planning processes and awareness of the potential of integration. While some proposed measures have already gone through environmental or strategic impact assessments, others contained a general statement of awareness of environmental laws and a pledge of full compliance with them at the stage of implementation.

The Commission’s evaluation of NRRPs and *do no harm* followed a standard structure of the report, the qualitative evaluations differed. The Commission was visibly more critical towards some countries (e.g. Bulgaria, Czech Republic, Finland, Portugal) for not doing enough to support green transition, including biodiversity, while not consistently criticizing those that designed no or very few measures targeting biodiversity (e.g. Estonia, Germany, Slovenia). Countries that did not dedicate any funds to biodiversity were not critiqued. The criticism does not seem to be linked to the scope or nature of funds and could be perhaps better explained by the opinion of the individual evaluator.

Several observations can be made regarding the impact of NRRPs on biodiversity. Firstly, most reforms and investments provide very little to no justification over how the conclusion that they have no significant impact on the biodiversity or any other environmental area was arrived at. System-wide implications along various sustainability dimensions, including biodiversity, were not deliberated or fully explored. An example is the non-problematic presentation of the German investment in the electrification of private vehicles, despite the concerns related to the extraction of components for batteries and batteries recycling (Dirnaichner et al. 2022). On several occasions, benefits for biodiversity were mentioned as a positive ‘side-effect’ stemming from other actions. This was typically the case with the rise of renewables in national policy mixes. Sometimes the link between actions and benefits was elusive and not justified, as in the case of the Cypriot diversification of the economy, which is expected to have a contribution to protecting biodiversity through developing touristic routes or through improving waste collection and recycling.

Secondly, some investments have biodiversity as their primary target, but their share is low. Both at the individual country levels and at the cumulative EU level, they are significantly lower than investments towards decarbonisation and do not reach the targets indicated in the EU Biodiversity Strategy. The Strategy spoke of biodiversity and nature-based solutions benefitting from “a significant proportion of the 25% of the EU budget dedicated to climate action” and recognised as “among the five most important fiscal recovery policies, which offer high economic multipliers and positive climate impact.” (European Commission 2020d) Table 1 details investments into biodiversity across the NRRPs. At the EU level, these have reached merely 0,00,000,088% of the value of NRRP.^3^ At a country level, they range between 10 and 0% of all funds, in comparison to a minimum of 37% funds dedicated for decarbonisation. About half of the countries have earmarked only 0–1% of the funds for measures with significant or primary objective of biodiversity protection. The share of funds seems to correlate positively with the clarity of visions on how these funds will be used and of the total benefits of biodiversity, i.e. the lower the percentage of funds earmarked for biodiversity, the less detail is given on their spending.

**Table 1:** Overview of the shares of NRRPs dedicated to biodiversity

Country	Does NRRP state it contributes to biodiversity?	Amount dedicated to biodiversity (in mio Eur)	Value of contribution to biodiversity as % of the total NRRP value %	Key measures addressing biodiversity
Austria	Yes	55	1.22	Biodiversity fund; climate-friendly town centres (green façades projects)
Belgium	Yes	399,66	6.74	Biodiversity and adaptation to climate change
Bulgaria	Yes	47,5	0.77	Ecosystem approach, restoring key climate systems
Croatia	Yes	33,1	0.52	Restoring rivers, floodplains and lakes
Cyprus	Yes	0	0.00	–
Czechia	Yes	117	1.65	Promotion of biodiversity; protection against drought
Denmark	Yes	0	0.00	Organic farming
Estonia	No	0	0.00	Vegetation of a new building; native species replanted
Finland	Yes	20	0.95	Nutrient recycling and forest management
France	Yes	335	0.82	“Biodiversity” unspecified
Germany	No	0	0.00	–
Greece	Yes	105	0.58	Revitalization of most affected areas unspecified
Hungary*	Yes	18,4	0.26	–
Ireland	Yes	108	10.92	Rehabilitation of peatlands to enhance biodiversity, ecosystem resilience and increase carbon sink
Italy	Yes	757	0.40	Restoration and protection of river and marine habitats
Latvia	No	0	0.00	Flood risk reduction
Lithuania	Yes	19	0.86	Peatland restoration
Luxembourg	Yes	6	6.67	Protection of environment unspecified
Malta	No	0	0.00	–
Poland*	No	0	0.00	–
Portugal	Yes	572	3.44	Prevention from forest fires, investment into more sustainable blue economy
Romania	Yes	1147	3.90	Afforestation, flood prevention, protected areas biodiversity strategy
Slovakia	Yes	159	2.43	Renaturation, protected areas development
Slovenia	No	0	0.00	Only indirect benefits mentioned
Spain	Yes	1730	2.49	Conservation, recovery and restoration
Sweden	Yes	247	7.48	Protection of forest reserves
**EU level**		**5875,66**	**1,2%**	

*NRRP not approved yet

Thirdly, not all the measures targeting climate mitigation or adaptation have considered benefits for biodiversity. Synergies between climate and biodiversity goals have inconsistently been identified and sometimes missed. For example, measures for flood prevention are sometimes not specified (Czech Republic, Latvia) and give reasons for concern over harming, instead of benefitting biodiversity. Synergies between climate and biodiversity policy, Nature-Based Solutions or Natural Climate Solutions as opportunities for maximizing social and ecological benefits and meet global development goals and cost-effective long-term solutions for sustainability have a significant potential in enhancing resilience and sustainability (Griscom et al. 2017; Keesstra et al. 2018; Palomo et al. 2021). The inability to encourage or even mandate such science-based solutions and targets is certainly the procedure’s weakness.

Fourthly, very few NRRPs introduce or commit to mitigation measures for the investments that have been selected for funding. Exceptions are the Estonian plan to build a hospital specifies that biodiversity will be protected by vegetating 50% of the hospital area and with the native species replanted, or Austrian commitment to setting sustainability standards for the projected rise in biomass. While countries may intend to mitigate the impacts at a later stage, through the Environmental Impact Assessments of individual projects, the *do no harm* procedure did not encourage them to make biodiversity an inherent part of the planned investments.

**Fig. 2:**
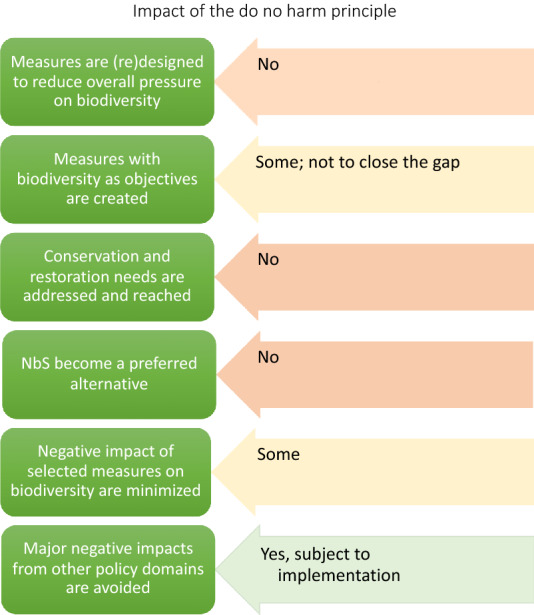
Impact of the *do no harm* on biodiversity outcomes

Lastly, major negative impact on biodiversity sensitive areas, which would result in manifest errors, have been avoided in the adopted NRRPs. Most controversial measures have been removed in the course of the adoption of plans (CEE Bankwatch Network 2021). However, civil society within countries remains concerned about the investments disregarding biodiversity goals, once they have received the green light for funding (CEE Bankwatch Network 2022). The confidence that harm will actually be avoided remains contingent on the scrutiny of implementation of NRRPs (Fig. 2).

## Discussion: *do no harm* as a viable policy innovation?

The *do no harm* represents a proactive policy measure for avoiding conflicts between various environmental measures at the final stages of the planning procedure. In comparison to existing tools for integration, the value-added by the *do no harm* is twofold. First, the procedure mandates, rather than just promotes a consideration of whether certain measures impact on biodiversity and other environmental goals. Secondly, it introduces a consideration of the interplay among policy measures across environmental policies to the *administrative* process, rather than the legislative level. By treating the administrative process as significant for effective regulation (Croley 1998), it addresses a frequently existing gap between intentions and reality.

*Do no harm* could be an incremental step in the process of changing attitudes and culture within government agencies and in the direction of more holistic thinking about the integration of environmental goals. This is a particularly important impact, considering the departments preparing investment plans were typically those grounded in economy or finance, rather than environment. Indeed, a result of the sensitizing policy-makers to the interplay among environmental targets may be seen in the fact that some of the controversial projects, rumoured to be proposed for recovery funds in the national circles, such as the Bulgarian plan of a large scale irrigation system and a hydro-melioration project that had not conducted any SEA (CEE Bankwatch Network 2021), have been excluded from the final NRRPs.

However, *do no harm* should not be seen as an effective means of operationalising integration of biodiversity and other environmental policies into the planning of investments and reforms supported by public money. As a policy instrument, it had limited chances for inducing coherence when this was a challenge at the levels of problem definition and policy formulation (Kurze and Lenschow 2018; Bocquillon 2018). Compared to climate change clearly taking a priority with its strong policy target (37% of investments), biodiversity had a less favourable vantage point. This was difficult to be surpassed by a weak procedural instrument, designed to take effect late in the process, which was already characterised by a tight schedule. Far from the ability to induce deliberation into policy-planning, stimulate or even instruct synergistic measures, its potential was limited to introducing a routine and lengthy self-care check-list, requiring little detail in justification and not providing for an effective external review, including that of the public.

The setup of *do no harm* effectively rendered the procedure a legitimising, rather than an evaluative instrument. For most member states, the preparation of NRRPs was motivated by the aim of completing it in time and designating money to intended projects, while complying with digital and climate targets. Quite to the contrary of stimulating the design of NRRPs and acting as a starting point of departure, *do no harm* was a validating errand before the adoption of plans in form of a relatively uncomplicated procedural requirement.

The *do no harm* did not go beyond the existing requirements of the environmental legislation in the EU (Jordan and Dupont 2021). Its overall effect on biodiversity mainstreaming was largely insignificant. Across the EU, *do no harm* did not reverse the trajectory of tackling biodiversity loss and climate change independently of each other (Pörtner et al. 2021). Neither did it set off the intended life-cycle thinking (European Commission 2021a) or desired imagination or deliberation process (Milkoreit 2017; Miller and Wyborn 2020; Hammond 2020). At worse, *do no harm* was misleading in terms of presenting biodiversity-positive efforts when these were not tangible, and validating institutional and technological lock-ins (Farla et al. 2012; Seto et al. 2016) as well as incumbent power structures (Stirling 2015).

**Fig. 3:**
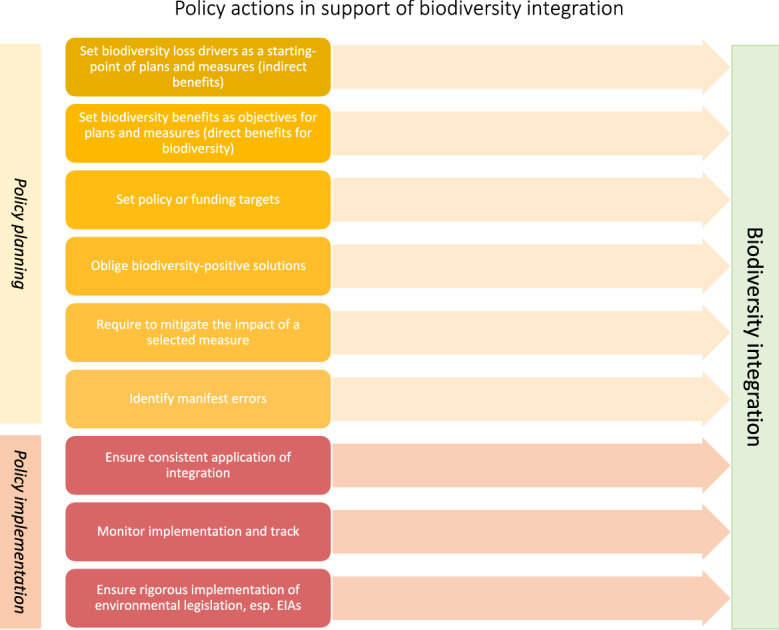
Regulatory actions in support of biodiversity integration

The success of the *do no harm* remains to be contingent on the implementation of the plans. Several measures remain relevant in this regard. First, it is important to ensure that the biodiversity-harmful projects, which were removed from being funded under the recovery EU funds, are not funded under other EU funding (such as cohesion funds, which do not yet require the application of *do no harm)* or national funding. Second, it would be necessary to monitor the investments indicated as ‘green’ and track their impact on the biodiversity, as a way of improving transparency and quantifiability of biodiversity (Deutz and et al. 2020). Third, there is scope for existing legislation, primarily a rigorous implementation of Environmental Impact Assessments, to play a role in mitigating the impact of selected measures on biodiversity (Biasotto and Kindel 2018). Regulatory actions at disposal of public authorities in support of biodiversity integration, both in policy planning and in policy implementation phase, and are listed in Fig. 3.

## Conclusion

In the attempt to expand knowledge on actions by public authorities for fostering change towards a sustainable world, this article considered a specific approach in channelling public investments. The *do no harm* principle was introduced in the EU in 2021 as a condition for receiving public funds from the post-Covid recovery stimulus package. It was envisaged as instrumental for the EU’s green transition and for enhancing the benefits for biodiversity. While *do no harm* attempted policy coherence and integration of policy goals, both of which are recognised as supportive of sustainability transformations, the approach mostly worked to maintain incumbent decision-making, where biodiversity conservation and its sustainable use do not represent policy objectives and are rarely fostered along the decision-making process. The greatest impact of *do no harm* procedure is likely to have been at the level of sensitizing administrative staff to a wider spectrum of environmental goals, while it cannot be linked to any policy outcomes in support of biodiversity.

The lack of impact of the *do no harm* principle on the biodiversity is particularly relevant in the era of a strong political rhetoric over the need for sustainability transformations. The EU’s experience from the Covid-19 recovery demonstrates that ‘green transition’ for the EU is largely conceived as a transition to decarbonization through technological investments that pay little attention to the concurrent need to preserve biodiversity and functioning ecosystems. At the level of the EU, the extent of investments and the level of political willingness towards climate neutrality is far from matched by the dedication to biodiversity.

The framing of the transformation as a challenge of switching energy sources and phasing out fossil fuels, including with suboptimal and harmful implications for biodiversity, presents a real challenge to the integration principle (Kurze and Lenschow 2018; Loorbach 2020). The ongoing failure of long-term-oriented and holistic cultures, structures and practices could not be solved through the introduction of the specific instrument of *do no harm*. This is not a critique of procedure-based instruments altogether: they are capable of complementing target-centred governance approaches. We have suggested a combination of measures to arrive at public deliberation and goal-setting, as well as more technical processes of tracking, verification and screening, as viable regulatory approach for public authorities’ integration of biodiversity targets. When improperly designed, regulation is capable of failing biodiversity, as the present case study has shown. But it also has a strong potential in devising (more ambitious) targets and encouraging and ensuring biodiversity considerations. While such actions by public authorities do not replace the significance of bottom-up initiatives and emerging alternative movements, they are an earnest strategy to enact positive action, rather than linger on the impossibility of change.

## Data Availability

The data is available with the author.

## Footnotes

1 The think tank Bruegel produced a dataset on the spending in recovery and resilience plans datasets in 2021 but it is currently unavailable. Green Recovery Tracker dataset was produced in 2021 and is available at https://www.greenrecoverytracker.org/. Neither dataset contained values for biodiversity funding. An economic impact analysis of 10 NRRPs, using impact modelling methodologies, was developed by Vivid Economics 2021.

2 UNCLOS Art 194 para 2; UNFCC recital 8 of the preamble; CBD Art 3; see also Rio Declaration principle 2.

3 According to an estimate, only about 8% of all spending tagged as “green” enhances nature (Vivid Economics 2021).
